# Optimization and automation of rapid and selective analysis of fatty acid methyl esters from aqueous samples by headspace SPME arrow extraction followed by GC–MS/MS analysis

**DOI:** 10.1007/s00216-022-04204-2

**Published:** 2022-07-19

**Authors:** Lucie K. Tintrop, Maik A. Jochmann, Thomas Beesley, Marco Küppers, Ruth Brunstermann, Torsten C. Schmidt

**Affiliations:** 1 Instrumental Analytical Chemistry, Faculty of Chemistry, University of Duisburg-Essen, Universitätsstraße 5, 45141 Essen, Germany; 2 Centre for Water and Environmental Research, University of Duisburg-Essen, Universitätsstrasse 5, 45141 Essen, Germany; 3 Urban Water and Waste Management, Faculty of Engineering, University of Duisburg-Essen, Universitätsstraße 15, 45141 Essen, Germany; 4 IWW Water Centre, Moritzstrasse 26, 45476 Mülheim an der Ruhr, Germany

**Keywords:** Fatty acid methyl esters, SPME arrow, GC–MS/MS, Bioreactor, Water

## Abstract

The analysis of fatty acid methyl esters (FAMEs) is of high relevance for monitoring and control of various industrial processes and biological systems. In this study, a novel, green analytical approach for the determination of 24 FAMEs from aqueous samples is proposed, which is based on a headspace solid-phase microextraction (SPME) arrow followed by gas chromatography coupled to tandem mass spectrometry (GC–MS/MS). The method was substantially accelerated to a run time of 44 min per sample by thorough optimization and automation of the relevant parameters. The limiting parameters, mostly based on expediting equilibrium attainment, were found to be parameters of extraction: material, pH, time, and temperature, which were optimized to divinylbenzene polydimethylsiloxane (DVB-PDMS), pH 2, 20 min, and 70 °C, respectively. The optimization and automation of the method led to low method detection limits (9–437 ng L^−1^) and high selectivity. Evaluation of the method on real samples was done by analyzing the aqueous phase of a bioreactor, whereby the matrix effect could be greatly reduced due to dilution and headspace sampling. The rapid, sensitive, selective, and matrix-reduced approach is found to be not only a novel method for water analysis but is promising for further applications, e.g., with solid and gaseous samples containing FAMEs.

**Graphical abstract:**

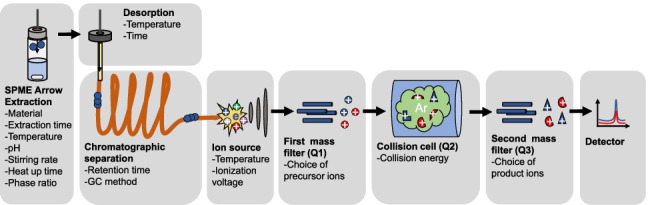

**Supplementary Information:**

The online version contains supplementary material available at 10.1007/s00216-022-04204-2.

## Introduction

Fatty acid methyl esters (FAMEs) are relevant substances in the food industry, microbiology, water analysis, and biodiesel production [1–4]. In these fields, they are mainly investigated for quality or process control and serve as major components in biodiesel [5], lipid metabolism products in plants and microorganisms [2], food ingredients [1], and as potential new antibiotics that could contribute to solving the problem of multi-drug resistance [6]. Furthermore, FAMEs serve as platform chemicals and are used for large-scale industrial production of surfactants, emulsifiers, or resins [7]. In industrial production, FAMEs are easier to handle in comparison to fatty acids, since they have the advantage of lower boiling points and are less corrosive [7]. The methyl esterification of fatty acids to FAMEs is a frequently used derivatization method in fatty acid analysis by GC. However, it is not possible to distinguish naturally occurring FAMEs from the derivatized fatty acids in the sample when esterification is applied. Therefore, it is important to determine the FAMEs in the sample prior to derivatization.

In Germany, the Renewable Energy Sources Act (EEG) stipulates that the percentage of renewable energy should be increased to 60% by 2035. Especially, carbon-rich wastewaters as feed for bioreactors have the potential of being reused as a basis for platform chemicals, whereby large amounts of carbon dioxide can be saved [8, 9]. Particularly, the direct extraction of FAMEs from such bioreactors is of interest as they can be utilized in biodiesel production.

The determination of FAMEs by gas chromatography (GC) has already been described using various detectors, including flame ionization detector (FID) [10], mass spectrometry (MS) [11], and tandem mass spectrometry (MS/MS) [5]. However, complex matrices require the extraction of FAMEs. Frequently used sample preparation methods to separate and enrich FAMEs from the matrix are solid-phase extraction (SPE) [12] and liquid–liquid extraction (LLE) [5] as well as microextraction methods such as SPME [13], hollow-fiber liquid-phase microextraction (HF-LPME) [14], and dispersive liquid–liquid microextraction (DLLME) [3]. In general, extensive solvent, time, and material-consuming LLE and SPE methods should be avoided for environmental reasons. This has been remedied by miniaturization of the extractions, which save solvents in the case of DLLME and HF-LPME and even operate solvent-free in the case of SPME. The development of SPME to SPME arrow reduced problems concerning mechanical stability and increased the size of the sorption phase [15]. After the first study on SPME arrow sampling in 2015 [16], its use has been described in many studies covering different analytes and applications, including determination of polycyclic aromatic hydrocarbons in water [15], organic compounds in the atmosphere [17], volatiles in fish sauce [18], amines in wastewater, and phosphorous flame retardants in water [19], among others.

Analytical chemistry has a major responsibility concerning the adaptation and development of green and sustainable methods. Green analytical chemistry does not clearly define the characteristics of a green method but increases awareness for miniaturization, automation, direct analysis, reagent replacement, multianalyte determinations, and operator safety, among others [20–22]. The demands for greener, faster, and more sensitive methods are ever-increasing. Automation, method optimization, solvent-free microextraction methods, and SPME arrow, in particular, offer a high potential to meet future demands and thus are worth further research and study [20, 23].

This work is the first to deal with SPME arrow extraction of 24 FAMEs from aqueous samples. SPME arrow is used as a green extraction method, which is solvent-free in contrast to conventional extraction methods and has a larger phase volume compared to SPME. Since the extraction parameters had a strong influence on the success of the method, this was intensively studied and discussed, which can provide the basis for future studies in this field. The novel combination of sensitive SPME arrow headspace extraction without matrix contact and selective determination by GC–MS/MS operating in MRM (multiple reaction monitoring) mode indicates an almost unlimited range of possible applications in target analysis.

## Material and methods

### Reagents and materials

Methyl heptanoate (C7:0Me, ≥ 99.8%), methyl nonanoate (C9:0Me, ≥ 99.8%), isotope-labeled methyl heptadecanoate-d_33_ (C17:0dMe, ≥ 97.5%), and 37-component FAME mix with varying concentrations of 200–600 mg L^−1^ were purchased from Sigma Aldrich (Steinheim, Germany). The FAME mix with varying concentrations was containing the following compounds (used abbreviations are given in brackets): methyl hexanoate (C6:0Me), methyl octanoate (C8:0Me), methyl decanoate (C10:0Me), methyl undecanoate (C11:0Me), methyl dodecanoate (C12:0Me), methyl tridecanoate (C13:0Me), methyl tetradecanoate (C14:0Me), methyl pentadecanoate (C15:0Me), methyl hexadecanoate (C16:0Me), methyl heptadecanoate (C17:0Me), methyl octadecanoate (C18:0Me), methyl eicosanoate (C20:0Me), methyl heneicosanoate (C21:0Me), methyl docosanoate (C22:0Me), methyl cis-9-hexadecenoate (C16:1cMe), methyl trans-9-octadecenoate (C18:1tMe), methyl cis-9-octadecenoate (C18:1cMe), methyl all-cis-9,12-octadecadienoate (C18:2cMe), methyl all-cis-6,9,12-octadecatrienoate (C18:3c6Me), methyl all-cis-9,12,15-octadecatrienoate (C18:3c9Me), and methyl all-cis-5,8,11,14,17-eicosapentaenoate (C20:5cMe). Sulfuric acid (H_2_SO_4_, ≥ 95%) was purchased from Fisher Scientific (Loughborough, UK). Stock solutions of single substances (1 g L^−1^) were prepared in methanol (100.0%, VWR, Fontenay-sous-Bois, France) and stored at 4 °C. Samples for the optimization and method validation procedure were prepared in bidistilled water (Bi-Distillation apparatus Bi 18E with quartz glass, Quarzglas QCS, Germany).

### GC–MS/MS analysis

Separation and detection were performed by a Shimadzu GCMS-TQ-2010 (Shimadzu Deutschland GmbH, Duisburg, Germany) in MRM scan mode equipped with a Zebron ZB-FAME capillary column (30 m × 0.25 mm × 0.20 µm, Phenomenex, Torrance, USA) using helium (5.0, AirLiquide, Oberhausen, Germany) as carrier gas with a column flow of 1.8 mL min^−1^ and argon (5.0, AirLiquide, Oberhausen, Germany) as collision gas. The oven temperature program started at 40 °C, was held for 5 min, and was raised with a rate of 6 °C min^−1^ to 210 °C. The injector, transfer line, and ion source temperature were set to 250 °C, 180 °C, and 180 °C, respectively. Thermal desorption of the analytes from the SPME arrow fiber was conducted in the injector for 4 min with a solvent cut time of 5 min. The GC injection port was modified for use of wider SPME arrow fibers and a splitless liner (1.8 mm × 5 mm × 95 mm, Topaz Liner, Restek, Bad Homburg, Germany) was installed. The optimized MRM settings can be seen in Electronic Supplementary Material (ESM) in Table S2 and the ionization voltage and emission current were 70 eV and 60 µA, respectively. The method was used for SPME arrow injection as well as liquid injection with 0.5 µL injection volume. As response, the peak area determined by the TIC of MRM transitions was used [24]. Two transitions are sufficient to identify compounds according to the European Commission [25], but to increase the robustness of identification and to extend the applicability of the method in future works, multiple transitions were optimized which can be used for the determination of FAMEs with GC–MS/MS operating in MRM mode. The geometric mean was used for averaging normalized data.

### Sample preparation, extraction, and automation

Since the analytes are extracted from the headspace, the ratio of headspace to liquid phase was 10 mL:10 mL. After heating the sample for 14 min (determined in pre-experiment), the SPME arrow fiber is inserted through the septum in the cap of the vial and the phase material is exposed. The FAMEs are sorbed to the phase material and after the extraction time has elapsed, the phase material is retracted into the cavity of the tool. The FAMEs are then immediately desorbed in the GC injector.

Full automation of the sample preparation, including the addition of standard solutions, pH adjustment, and extraction, was conducted with an RTC PAL autosampler and the following units: Agitator, DeCapper module, FiberConditioning module, Wash station, SPME arrow tool, 10 µL liquid tool, 100 µL liquid tool, and tool park station, all from CTC Analytics (Zwingen, Switzerland) and heating and stirring plate RTC basic (IKA, Staufen, Germany). SPME arrows were used with polydimethylsiloxane (PDMS), divinylbenzene polydimethylsiloxane (DVB-PDMS), carbon wide range divinylbenzene polydimethylsiloxane (CWR-DVB-PDMS), carbon wide range divinylbenzene (CWR-DVB), and polyacrylate (PA) as polymer sorption phases (BGB Analytik, Böckten, Switzerland).

The workflow for one sample was as follows: Sample is opened (DeCapper module) and standard solutions and H_2_SO_4_ for pH adjustment are added (liquid tools); tempering of MeOH-vial and chemical fiber cleaning in the headspace of MeOH (Heatex); thermal fiber cleaning (Fiber conditioning module); tempering and stirring (heating and stirring plate); extraction (SPME arrow tool); and desorbing in GC injector (SPME arrow tool). The autosampler and automation software used was Chronos (version 5.1.20, Axel Semrau, Strockhoevel, Germany). The total method run time is 82 min. Due to an overlapping schedule, the next sample can already be prepared while the GC–MS/MS measurement is running. This saves 38 min per sample and results in a reduced run time of 44 min for one sample.

### Optimization procedure

Selection of the optimal extraction parameters was done by one-factor-at-a-time (OFAT) and design of experiment (DOE) using the design of experiments application of OriginPro (Version 2020, OriginLab Corporation) [26]. The use of DOE reduces the sample number to a minimum, saves resources, and additionally observes factor interactions. The DOE model used was a Box-Behnken model with the following settings: center points per block: 1, randomized design.

### Method validation

#### Analytical performance

The calibration standards were prepared by spiking bidistilled water, acidified to pH 2, with the FAME mix with varying concentrations. The internal standard (C17:0dMe) was spiked to each sample of method validation in a concentration of 2 µg L^−1^ and the peak areas of each analyte were normalized to the internal standard peak area. As the calibration ranged over two orders of magnitude, two concentration ranges were chosen for calibration. In the first calibration, the concentration of analytes ranged from 10 to 1,500 ng L^−1^ with five concentration levels and in the second calibration, the concentration ranged from 500 to 10,500 ng L^−1^ (or 0.5–10.5 µg L^−1^) with seven concentration levels. For each concentration level, 7 replicates were measured, leading to the relative standard deviation (RSD) values. To further evaluate the method, the method detection limit (MDL) was determined according to US environmental protection agency’s MDL procedure (Revision 2, 2016) [27]. The MDL was obtained at a concentration level with an S/N ratio of 5 to 1 for all FAMEs in a spiked blank sample by using the single-tailed Student’s *t*-value with a confidence level of 99% (${t}_{(6, 1-\alpha = 0.99)}=3.143)$ and the standard deviation ${S}_{s}$ of seven replicates (${MDL}_{s}={t}_{(n-1, 1-\alpha = 0.99)}{S}_{s}).$

#### Extraction efficiency

To evaluate the efficiency of extraction, the depletion curve method according to Zimmermann et al. was used [28]. To that end, the sample is extracted 10 times for determining the depletion of the analyte’s peak area, which in general is following a model function for exponential decay $f(x) = {ab}^{x}$. After that, the depletion curve is linearized by applying the natural logarithm (ln) to the peak area; consequently, the depletion is following the model function for linear equations $f(x) = a + bx$. From the linear equation, the extraction efficiency ${E}_{E}$ is obtained as the slope $b$ of the linear equation by $1-{E}_{E}={e}^{b}$.

#### Real sample

The real sample was taken from the aqueous phase of a bioreactor feed by carbon-rich wastewater from a combustion plant. The quantification of the FAMEs was done by diluting the sample 1:4, 1:10, 1:100, and 1:1,000, and normalizing the peak area to the internal standard. Relative recovery of the FAMEs in the real sample was obtained by dividing the 1:100 diluted sample into 10-mL aliquots to allow the measurement of triplicates of the spiked and non-spiked sample. The FAME mix with varying concentrations was spiked to the sample to achieve a dilution of 1:400,000 (corresponds to concentrations of 0.5–1.5 µg L^−1^). Relative recovery was calculated by subtracting the spiked concentration from the non-spiked concentration and matching the result with the calibration.

## Results and discussion

### GC–MS/MS analysis

**Fig. 1:**
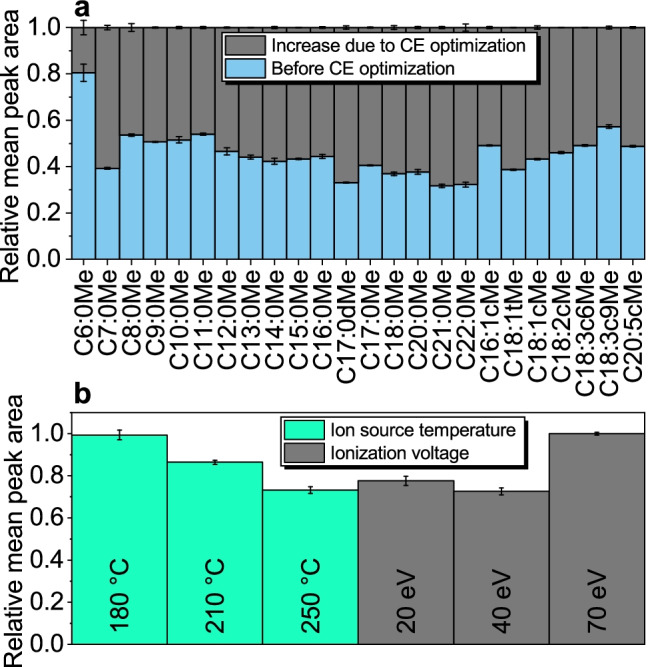
Results of GC–MS/MS FAMEs method optimization. **a** Relative peak areas before (blue) and increase after collision energy optimization (grey). **b** Optimization of ion source temperature (green) and ionization voltage (grey). Experimental conditions: *n* = 3; **a** CE before optimization: 5 eV, CEs for optimization: 5, 10, 15, 20, 25, and 30 eV; **b** ionization voltage at ion source temperature optimization: 70 eV, ion source temperature at ionization voltage optimization: 250 °C

Since the electron impact (EI) ion source strongly fragments the FAMEs, the molecular ion has a low abundance leading to lower sensitivity when exclusively used as precursor ion in MRM. Therefore, in addition to the molecular ions, specific EI fragments, previously selected by a product ion scan and according to Härtig et al. [29] (see ESM Table S1), were chosen for the MRM. These smaller fragments are generated in the EI ion source and can be assigned to a substance by chromatographic separation in combination with the previous product ion scan. The selection of precursor and product ions is shown in ESM in Table S2. In this work, all 5–6 transitions were used as quantifiers and qualifiers. Using the here-stated MRM transitions and the TIC of MRM transitions for quantification may not apply to every sample matrix due to possible interfering compounds. Therefore, its use has to be evaluated in method development by observing the ion ratios. Although the use of TIC of MRM transitions for quantification could lead to higher error proneness, on the other hand, it gains sensitivity so that the matrix can be diluted and dilution can reduce matrix effects. The deviation of the ion ratios was monitored throughout the measurements and was found to be always below ± 10% (see ESM Fig. S1) in ultrapure water and real sample and no errors and/or disturbing substances were found. The observed ion ratios for every analyte are shown in ESM in Table S2. To increase the response, the collision energy (CE) was optimized in the range of 5–30 eV, which is specific for each transition. The optimization resulted in a response increase of 20–60% (Fig. 1a). An ion source temperature of 180 °C and standard ionization voltage of 70 eV led to the highest response (Fig. 1b), as the abundance of the chosen fragments and molecular ions increased.

### Optimization of the extraction procedure

#### Effect of extraction temperature

**Fig. 2:**
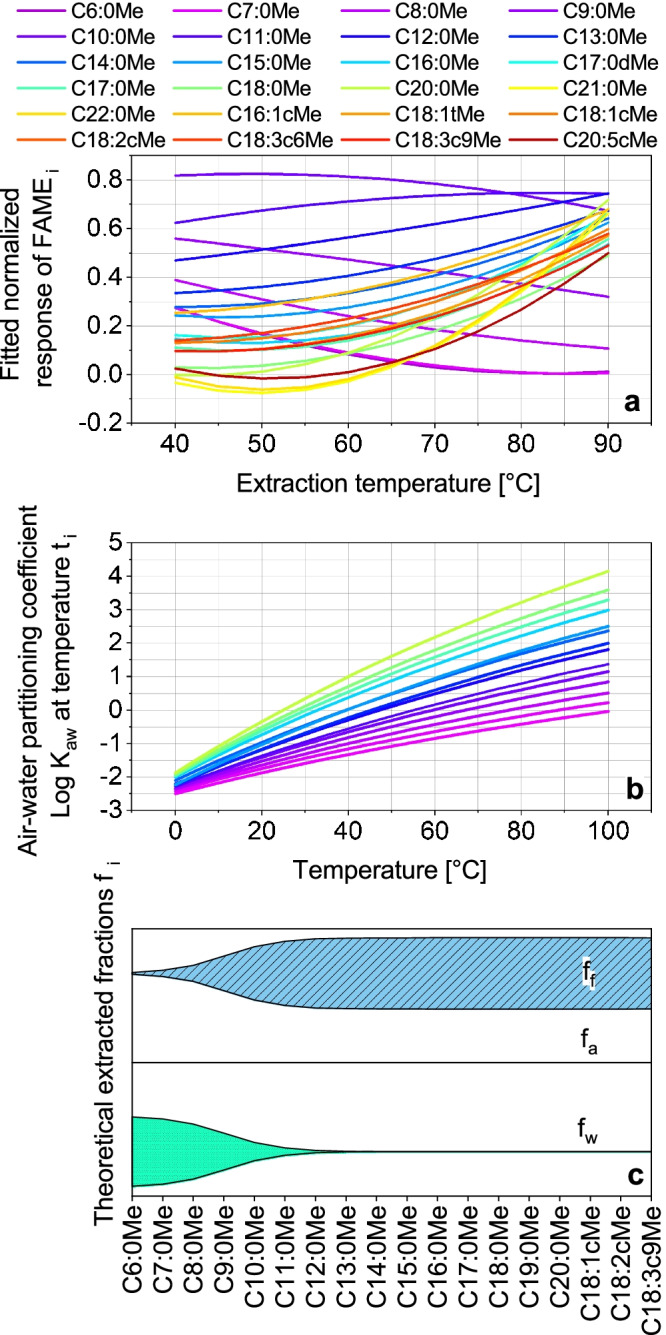
Results of extraction temperature optimization. **a** Fitted normalized response of extraction temperature dependency for single FAMEs obtained with DOE. **b** Temperature dependency of the Henrys law constant *K*_aw_ for saturated FAMEs. **c** Kite-plot of theoretical distributions of FAMEs in the three sample phases air *f*_a_, water *f*_w_, and sorbed to the SPME arrow fiber *f*_f_ (PDMS) at 25 °C for saturated FAMEs and some unsaturated FAMEs. Experimental conditions for **a**: *n* = 3; sample: FAME mix with varying concentrations 1:400,000 diluted in bidistilled water. Extraction parameters: stirring rate: 1,500 rpm, 30 min, DVB-PDMS, varying temperature (40–90 °C), pH 2

In order to optimize the extraction parameters, OFAT (time, material, cleaning, stirring rate) and DOE (temperature, pH) strategies were used. As a result of DOE, the normalized fitted response of temperature-dependent extraction yields for all FAMEs is shown in Fig. 2a (*R*^2^ = 0.9231 and adjusted *R*^2^ = 0.6922 of quadratic fit). At a higher temperature, the headspace concentration of the analytes increases (Fig. 2b, calculation shown in ESM; literature data not available for unsaturated FAMEs) consequently the extracted amount increases. Considering the three-phase system (aqueous phase, headspace, and fiber) and total equilibrium, the fraction of analytes located on the fiber is high for the > C13:0Me FAMEs (Fig. 2c), whereas the fractions in water and headspace are lower. The equilibrium constants were obtained with pp-LFER calculations (shown in ESM), but literature data were not available for all FAMEs and only for PDMS fiber material [30]. The relationship between high affinity to the phase material and the transfer of the analytes into the headspace leads to the effect that under equilibrium conditions, almost 100% of the > C13:0Me FAMEs can be extracted (Fig. 2c, see ESM Table S3). Nevertheless, equilibrium is often not reached during sample preparation due to time constraints, resulting in lower fractions in practice. A different effect is revealed by the short-chain FAMEs (< C10:0Me), where the highest response is between 40 and 65 °C. This effect can be explained by the low affinity to the phase material combined with an accelerated sorption/desorption equilibrium for the < C10:0Me FAMEs.

A higher extraction temperature leads to increased droplet formation due to the condensation of water on the SPME arrow material (see ESM Fig. S4) and an increase of retention time, only determined for C6:0Me, averaged to 7.15 min at 40 °C, 7.18 min at 65 °C, and 7.21 min at 90 °C. These retention time shifts are a sign of an increased amount of injected water since water expands a thousandfold in the injector and creates a thin layer inside the column [31]. The extraction temperature was lowered to 70 °C for the final method.

#### Effect of extraction pH

**Fig. 3:**
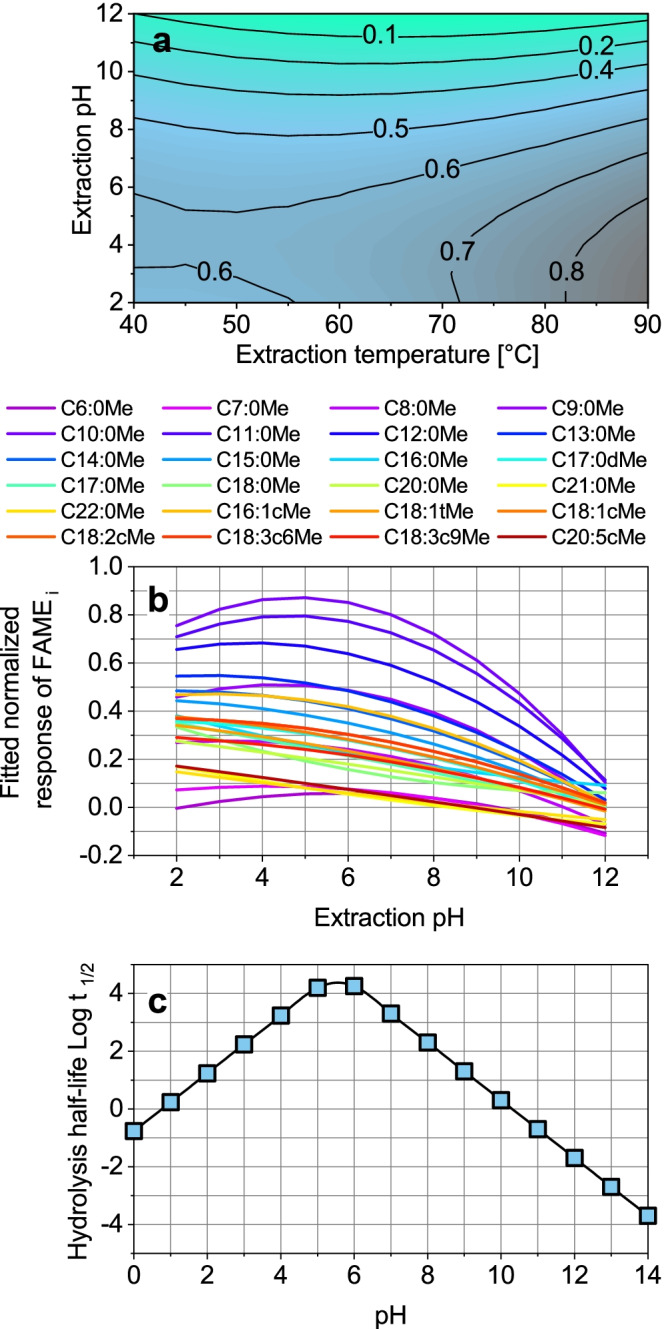
Results of extraction pH optimization. **a** Contour plot of extraction pH and extraction temperature optimization for all FAMEs. **b** Fitted normalized response of extraction pH dependency of single FAMEs. **c** Determined pH dependency of hydrolysis half-life *t*_1/2_ in days representative for all saturated FAMEs. Experimental conditions for **a** and **b**: *n* = 3; sample: FAME mix with varying concentrations 1:400,000 diluted in bidistilled water; extraction parameters: stirring rate: 1,500 rpm, 30 min, DVB-PDMS, varying temperature (40–90 °C), and pH (2–12)

To evaluate the effect of pH on hydrolysis of the FAMEs, the hydrolysis half-life is calculated with help of the pseudo-first-order rate constants at 25 °C for the acid-, neutral-, and base-catalyzed hydrolyses of saturated FAMEs, taken from Rayne et al. [32] (calculation shown in ESM). Results of Fig. 3a and b were obtained with DOE (*R*^2^ = 0.9231 and adjusted *R*^2^ = 0.6922 of quadratic fit). At 40 °C, the optimal pH value is between 3 and 6, which corresponds to a hydrolysis half-life above 100 d (Fig. 3a and c). At 90 °C, however, the optimal range of pH value shifts to pH 2, indicating acid-catalyzed hydrolysis must have a negligible influence on response reduction. Literature data for Fig. 3c was not available for unsaturated FAMEs and literature data for temperature-dependent hydrolysis half-life was also not available. Figure 3c shows the regular hydrolysis half-life shape with a maximum hydrolysis half-life at pH 5.5 and a minimum hydrolysis half-life at pH 14. Thus, the base-catalyzed hydrolysis is three orders of magnitude faster and more relevant than the acid-catalyzed hydrolysis. The stronger effect of base-catalyzed hydrolysis can be confirmed, resulting in a 50% response reduction from pH 6 to 12 (Fig. 3a and b). When the effect of pH on the individual FAMEs is considered (Fig. 3b), the optimal pH of 2 is confirmed in most cases but is almost constant between pH 2 and 5 for < C12:0Me FAMEs. To keep the effect of hydrolysis time as low as possible, the pH adjustment of every single sample was done by the autosampler directly before sample heat up, avoiding any storage time.

#### Effect of extraction time

**Fig. 4:**
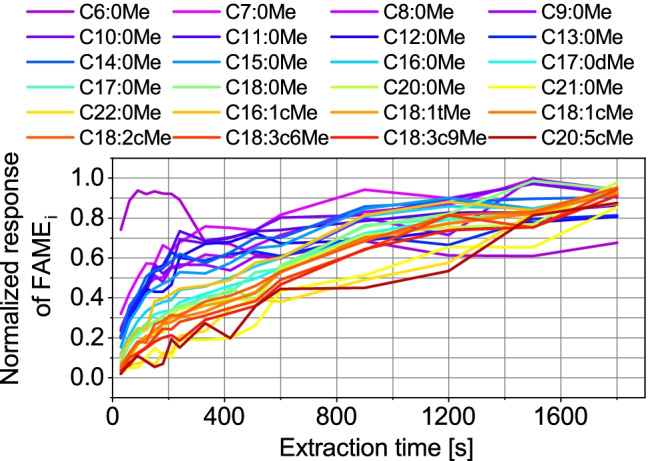
Results of extraction time optimization showing extraction profiles for single FAMEs. Experimental conditions: *n* = 3; sample: FAME mix with varying concentrations 1:400,000 diluted in bidistilled water; extraction parameters: stirring rate: 1,500 rpm, varying extraction time (30, 60, 90, 120, 150, 180, 210, 240, 330, 420, 510, 600, 900, 1,200, 1,500, 1,800 s), DVB-PDMS, 70 °C, pH 2

To determine an averaged optimal extraction time, experiments were performed with 30 to 1,800 s extraction time, with the longest extraction time 1,800 s (30 min) achieving the best results (Fig. 4). However, considering the benefit of a 10 min shorter method and a loss of only approximately 10% response, an extraction time of 1,200 s (20 min) was used in further experiments. Single extraction profiles can be found in the ESM (Figs. S2 and S3). For C6:0Me, the response remains constant (± 15%) during all extraction times due to fast equilibrium attainment, which can also be seen in the extraction profile for C6:0Me (see ESM Fig. S2).

#### Effect of extraction material

**Fig. 5:**
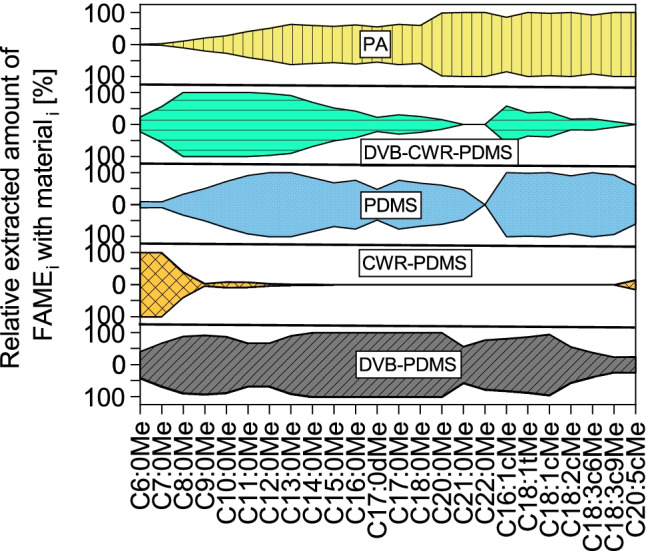
Results of SPME arrow material optimization presented as kite diagrams showing the distributions of the respective FAMEs on the different extraction phases. Experimental conditions: *n* = 3; sample: FAME mix with varying concentrations 1:400,000 diluted in bidistilled water; extraction parameters: stirring rate: 1,500 rpm, 30 min, varying SPME arrow material (PA, DVB-CWR-PDMS, PDMS, CWR-PDMS, DVB-PDMS), 70 °C, pH 2

Five SPME arrow sorbents PA, DVB-CWR-PDMS, PDMS, CWR-PDMS, and DVB-PDMS were tested (Fig. 5), with DVB-PDMS covering the broadest and CWR-PDMS the narrowest range of analytes. PDMS and PA are the best sorbents for unsaturated FAMEs, plus PA also covers long-chain FAMEs > C13:0Me. DVB-CWR-PDMS covers medium-chain C8:0Me-C12:0Me and CWR-PDMS only short-chain C6:0Me-C8:0Me FAMEs sufficiently well. To cover the broadest range of analytes, DVB-PDMS was used in the final method.

#### Effects of stirring rate, SPME arrow cleaning parameters, and stir bar material

Testing stirring rates of 500, 1,000 and 1,500 rpm for the extraction, the response was increased by 28% from 500 to 1,500 rpm (data not shown) due to faster equilibrium attainment. Since the analytes have a high affinity for the phase material, the carryover of the analytes into the next measurement has to be monitored. The correlation between carryover and cleaning process was determined by measuring blanks after each cleaning. The percentages for carryover refer to the quantity found in the blank in relation to the quantity in the previously measured sample. Different SPME arrow cleaning methods were tested, but the best results were achieved by a chemical cleaning step prior to thermal cleaning, which, to the best of our knowledge, has not yet been reported in literature. By adding a chemical cleaning step of the fiber in the headspace of MeOH (1 mL MeOH in 20 mL vial) for 2 min prior to the thermal cleaning step, the carryover could be reduced from > 4% (thermal cleaning) to < 1% (chemical and thermal cleaning), and additionally, the fiber cleaning parameters could be decreased from 15 min at 300 °C to 10 min at 280 °C. The vial for chemical cleaning can be reused several times (average 40 times in this study), depending on the used concentration. Without cleaning, 6% of the analytes are remaining on the fiber. Consequently, 94% of the analytes are desorbed in the injection. Longer-chain FAMEs > C16:0Me had a higher occurrence in the carryover because they have a higher affinity for the phase material and thermal desorption is slower. Another source of 8% analyte loss was obtained when Teflon instead of glass was used as stir bar material (data not shown). Therefore, glass stir bars were used in the following experiments.

### Validation of the analytical method

The FAMEs were calibrated in a range of 10–10,500 ng L^−1^. As this wide range showed different linearities, it was divided into two calibrations for the quantification calculations of the real samples. The results for both calibrations 10–1,500 ng L^−1^ and 500–10,500 ng L^−1^ are listed in Table 1. The low calibration showed good linearity for most of the FAMEs (*R*^2^ ≥ 0.9319); however, was not linear for C20:0Me, C21:0Me, C22:0Me, and C20:5cMe (*R*^2^ ≤ 0.8448). The non-linearity of the longer-chain FAMEs at the low calibration indicates that a fewer amount is injected into the GC, which could be due to less optimal extraction parameters. In addition, the sorption/desorption equilibrium attainment is slower and the FAMEs would need longer to increase the amount sorbed to the fiber (see time-dependent extraction profiles in Fig. 4). The high calibration showed good linearity for all FAMEs (*R*^2^ ≥ 0.9657). The MDLs ranged from 9 to 437 ng L^−1^ and were higher for long-chain FAMEs (≥ C18:0Me carbon atoms). Single calibration plots and linear regression functions of calibration are shown in the ESM (Figs. S5, S6, and Table S4). Extraction efficiencies ranged from 13 to 59% (*n* = 3, *R*^2^_E_ ≥ 0.7581), which are comparable with extraction efficiencies determined with SPME arrow extraction for PAHs by Kremser et al. [15]. A relative recovery of 69–127% was observed for spiked diluted (1:100) real samples in the dilution of 1:400,000 (corresponds to concentrations of 500–1,500 ng L^−1^). Single depletion curves and linear regression functions of extraction efficiency are shown in the ESM (Figs. S7, S8, and Table S4).

**Table 1:** Analytical method validation results. *R*^2^_1_, linear regression coefficient of calibration (10–1,500 ng L^−1^); *R*^2^_2_, linear regression coefficient of calibration (500–10,500 ng L^−1^); *MDL*, method detection limit; *RSD*_1_, relative standard deviation of calibration (10–1,500 ng L^−1^) at second calibration point (*n* = 7, 100–300 ng L^−1^); *RSD*_2_, relative standard deviation of calibration (500–10,500 ng L^−1^) at fifth calibration point (*n* = 7, 6,500–7,500 ng L^−1^); *E*_*E*_, extraction efficiency; *R*, relative recovery of analytes in 1:100 diluted real sample spiked with 500–1,500 ng L.^−1^; *c*, calculated concentration of analytes in real sample (diluted 1:4, 1:10, 1:100, and 1:1,000). Experimental conditions: optimized parameters were used

FAME	*R*^2^_1_	*R*^2^_2_	MDL [ng L^-1^]	RSD_1_[%]	RSD_2_[%]	${E}_{E}$ [%]	*R* [%]	*c* [µg L^−1^]
C6:0Me	0.9963	0.9761	23	31	27	13	75	27
C7:0Me	0.9606	0.9952	41	19	19	32	79	4
C8:0Me	0.9861	0.9992	16	11	15	59	76	0.3
C9:0Me	0.9883	0.9904	17	7	10	49	118	nd
C10:0Me	0.9962	0.9877	16	8	10	50	79	nd
C11:0Me	0.9999	0.9907	11	13	14	47	126	nd
C12:0Me	0.9966	0.9972	16	19	16	47	87	0.1
C13:0Me	0.9948	0.9953	13	23	16	48	127	nd
C14:0Me	0.9954	0.9875	26	19	9	47	88	1.3
C15:0Me	0.9933	0.9954	12	22	29	44	112	nd
C16:0Me	0.9862	0.9845	89	17	33	35	117	76
C17:0Me	0.9943	0.9793	10	24	36	20	117	11
C18:0Me	0.9829	0.9872	163	24	25	12	115	525
C20:0Me	nl	0.9818	119	nl	17	22	69	15
C21:0Me	nl	0.9657	124	nl	22	24	82	3
C22:0Me	nl	0.9928	290	nl	26	20	87	15
C16:1cMe	0.9878	0.9957	9	18	19	50	109	nd
C18:1tMe	0.9319	0.9755	14	37	36	22	109	nd
C18:1cMe	0.9664	0.9756	18	28	36	26	75	3490^a^
C18:2cMe	0.9758	0.9838	145	bm	32	42	100	402
C18:3c6Me	0.9622	0.9973	12	23	18	47	89	nd
C18:3c9Me	0.9503	0.9939	9	28	21	48	79	nd
C20:5cMe	nl	0.9817	437	bm	27	36	126	nd

^a^Was used as an extractant for the products in the bioreactor

*nd*, Substance is not determined; *nl*, calibration curve is not linear; *bm*, calibration point is below MDL

### Real sample analysis

**Fig. 6:**
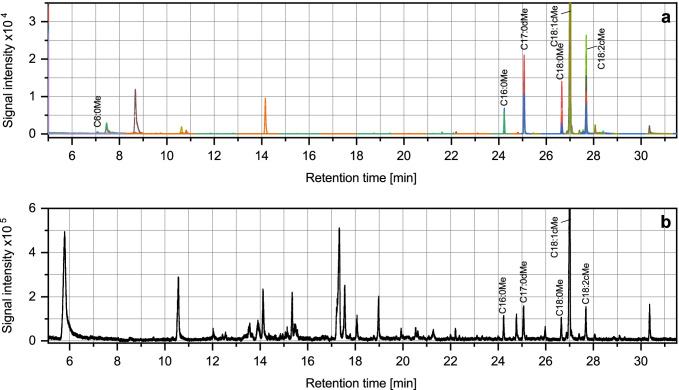
Chromatograms of real sample (1:100 diluted) from a bioreactor analyzed in MRM Mode **a** and Q3 Scan **b**. Colors indicate specific transitions of MRM according to Table S2

Analysis of the aqueous phase in a bioreactor showed the highest concentration for C18:1cMe (3.5 mg L^−1^), which was added to the bioreactor for extraction of the produced substrates. Additionally detected FAMEs, listed in Table 1, were in the range of 0.1–525 µg L^−1^. Figure 6 shows chromatograms of the optimized MRM (a) and a Q3 Scan (single quadrupole operation mode) of the sample (b). The baseline noise and interfering peaks could be eliminated using MRM. Additionally, more FAMEs, especially in low concentrations, could be detected by MRM.

### Comparison with other methods

To the best of our knowledge, there is no other study dealing with the extraction of FAMEs from aqueous samples via SPME arrow. Studies from the last 10 years proposed the analysis of FAMEs from biodiesel, wastewater, bio-liquid, and bacterial cells (after fatty acid derivatization) with LLE [5], DLLME [3], HF-LPME [14], SPE [12], and SPME [13]. Since biodiesel matrices differ widely from aqueous matrices, only the method for FAME determination in wastewater by Yu et al. is comparable with the samples presented here. The method proposed by Yu et al. was applied only to C16:0Me and C18:0Me and calibrated in a higher concentration range (0.2–5 mg L^−1^), exhibiting LODs of 0.05–0.3 mg L^−1^ and RSDs from 6 to 14%. The method proposed here covered 24 FAMEs and was calibrated in a lower concentration range to achieve matrix dilution of the samples. The LODs were lower and ranged from 0.01 to 1.9 mg L^−1^; however, the RSDs had a broader range of 7–37%.

## Conclusions

A fully automated and rapid green sample preparation and GC–MS/MS MRM method for the detection of FAMEs in aqueous samples was developed. Optimization and full automation greatly improved the analytical performance of the method. The optimal conditions depend on the structure of the FAME under consideration and can be compared and explained using theoretically calculated and literature data. Due to full automation and an overlapping schedule, 32 samples can be analyzed in 1 day (method run time 44 min). The sensitivity of the application was improved significantly by using SPME arrow but shows a smaller linear range and higher RSD values compared to conventional extraction methods. However, the vast majority of studies have been conducted analyzing high concentrations of FAMEs in non-aqueous samples (e.g., biodiesel), and methods dealing with the analysis of FAMEs in aqueous samples are still lacking. The results found in this study suggest that the method can be used for the analysis of FAMEs in samples even with high matrix load, with the advantages of MRM scanning, sample dilution, and headspace extraction. After this, further sample types (solid, gaseous, biological, etc.) for FAMEs analysis could be tested, wherein the method parameters of this study can be subsequently utilized.

## Supplementary Information

Below is the link to the electronic supplementary material.

Supplementary file1 (PDF 2.32 MB)
