# Supplementary material for: Optimization and automation of rapid and selective analysis of fatty acid methyl esters from aqueous samples by headspace SPME arrow extraction followed by GC–MS/MS analysis
Source: Anal Bioanal Chem. 2022 Jul 19;414(22):6473–83. doi: 10.1007/s00216-022-04204-2 (PMC9411252; doi:10.1007/s00216-022-04204-2)
Supplement: Supplementary file 1 — Supplementary file1 (PDF 2.32 MB) [file 216_2022_4204_MOESM1_ESM.pdf]

Electronic Supplementary Material for manuscript submitted to **Analytical and Bioanalytical Chemistry**

Optimization and automation of rapid and selective analysis of fatty acid methyl esters from aqueous samples by headspace SPME arrow extraction followed by GC-MS/MS analysis

Lucie K. Tintrop[a,b], Maik A. Jochmann*[a,b], Thomas Beesley[a], Marco Küppers[d], Ruth Brunstermann[d], Torsten C. Schmidt[a,b,c]

[a]Instrumental Analytical Chemistry, Faculty of Chemistry, University of Duisburg-Essen, Universitätsstraße 5, 45141 Essen, Germany

[b]Centre for Water and Environmental Research, University of Duisburg-Essen, Universitätsstrasse 5, 45141 Essen, Germany

[c]IWW Water Centre, Moritzstrasse 26, 45476 Mülheim an der Ruhr, Germany

[d]Urban Water- and Waste Management, Faculty of Engineering, University of Duisburg-Essen, Universitätsstraße 15, 45141 Essen, Germany

*Corresponding author (maik.jochmann@uni-due.de)

Table of Contents

1 Mass spectral fragmentation patterns 1

2 Optimized MRM parameters 1

3 Ion ratio stability 6

4 Theoretical extracted fractions 6

5 Single extraction profiles obtained by extraction time optimization 9

6 Calculation of hydrolysis half-life 11

7 Observation of droplet formation on SPME arrow surface 11

8 Calibration plots and linear regression functions 12

9 Depletion curves obtained by extraction efficiency experiments 15

References 17

1 Mass spectral fragmentation patterns

Table S1 General mass spectral fragmentation patterns for identification of FAMEs adapted from Härtig et al. [1]. Precursors and product ions were selected based on known fragmentations. M: Molecular ion

Fragments [m/z]	Identification
43/57/71/85	Alkyl series
41/55/69/83	Alkenyl series
59	Methoxy carbonyl
74	McLafferty rearrangement ion
75	Dimethoxy methyl radical ion
87	C3:0 Methyl ester
90	Cleavage at OH, H-rearrangement
103	Cleavage at OH
143	C7:0 methyl ester
199	Cleavage at C10 methyl branching site
M-15	Loss of methyl
M-18	Loss of water
M-29	Loss of ethyl
M-31	Loss of methoxy
M-32	Loss of methanol
M-43	Loss of propyl
M-46	Loss of ethyl + water
M-59	Loss of methoxy carbonyl
M-74	Loss of Mc Lafferty fragement

2 Optimized MRM parameters

Table S2 MRM transitions from precursor to product ions with optimized collision energy and ion ratios for the determination of FAMEs. Experimental conditions: Sample: FAME mix with varying concentrations (diluted 1:100 in acetone); n = 3; Chosen CEs for optimization were 5, 10, 15, 20, 25 and 30 eV. CE before optimization was 5 eV for all transitions. All transitions were used for quantification (TIC of MRM transitions) and qualification. Time frame: chosen time frame for the given transitions; *Molecular ions; CE: Collision energy

FAME	Abbr.	CAS No.	Retention time (time frame) [min]	MRM Transitions Precursor ions > Product ions [*m/z*]	Optim. CE [eV]	Ion ratios [%]
Methyl hexanoate	C6:0Me	106-70-7	7.160 (5.00-8.25)	130*>74	10	2
				130*>55	15	1
				99>43	20	51
				99>71	5	100
				99>59	10	5
Methyl heptanoate	C7:0Me	106-73-0	9.572 (8.25-11.20)	144*>115	10	0.1
				112>85	5	66
				112>43	10	100
				102>59	10	45
				102>74	10	65
Methyl octanoate	C8:0Me	111-11-5	11.751(11.20-13.00)	158*>74	15	2
				158*>101	5	6
				126>57	10	100
				126>109	5	10
				116>59	10	80
				116>83	10	8
Methyl nonanoate	C9:0Me	1731-84-6	13.700 (13.00-14.75)	172*>101	10	14
				172*>143	5	10
				140>71	5	45
				140>57	10	79
				130>69	10	100
				130>73	10	62
Methyl decanoate	C10:0Me	110-42-9	15.492 (14.75-16.5)	186*>101	5	8
				186*>129	10	8
				155>71	5	27
				155>57	15	29
				143>83	10	100
				143>87	10	21

Methyl undecanoate	C11:0Me	1731-86-8	17.176 (16.50-18.00)	200*>143	5	36
				200*>101	10	47
				169>95	5	72
				169>71	10	62
				157>97	10	100
				157>69	15	53
Methyl dodecanoate	C12:0Me	111-82-0	18.734 (18.00-19.50)	214*>143	5	20
				214*>157	5	20
				171>101	10	100
				171>69	10	69
				183>95	5	22
				183>109	5	21
Methyl tridecanoate	C13:0Me	1731-88-0	20.209 (19.50-21.00)	228*>143	10	21
				228*>157	5	16
				197>95	5	14
				197>71	10	22
				185>101	10	100
				185>69	15	46
Methyl tetradecanoate	C14:0Me	124-10-7	21.615 (21.00-22.10)	242*>157	5	15
				242*>171	5	13
				199>97	10	11
				199>101	10	93
				143>87	10	20
				143>83	10	100
Methyl pentadecanoate	C15:0Me	7132-64-1	22.947 (22.10-23.70)	256*>171	5	36
				256*>199	5	36
				213>107	10	6
				213>69	15	49
				157>101	10	100
				157>97	10	57
Methyl hexadecanoate	C16:0Me	112-39-0	24.238 (23.70-24.50)	270*>199	5	45
				270*>185	5	41
				238>109	5	9
				238>137	5	6
				171>101	10	100
				171>69	15	77
Isotope-labelled methyl	C17:0dMe	1219804-81-5	25.066 (24.95-25.40)	317*>219	10	69
				317*>171	10	32
				203>107	10	94

heptadecanoate-d33				155>94	10	100
				155>78	15	53
Methyl heptadecanoate	C17:0Me	1731-92-6	25.454 (25.40-25.80)	284*>199	10	43
				284*>143	10	43
				185>101	10	100
				185>83	10	18
				241>129	10	5
				241>97	10	13
Methyl octadecanoate	C18:0Me	112-61-8	26.657 (25.80-26.80)	298*>143	10	41
				298*>185	10	29
				255>109	10	6
				255>129	10	4
				199>101	10	100
				199>83	10	21
Methyl eicosanoate	C20:0Me	1120-28-1	28.855 (28.80-29.10)	326*>171	10	57
				326*>185	10	50
				283>101	10	100
				283>115	10	6
				227>111	10	7
				227>97	10	11
Methyl heneicosanoate	C21:0Me	6064-90-0	29.914 (29.10-30.20)	340*>185	10	78
				340*>143	10	70
				297>109	10	11
				297>83	15	19
				241>101	10	100
				241>85	10	6
Methyl docosanoate	C22:0Me	929-77-1	30.924 (30.20-31.35)	354*>199	10	73
				354*>143	15	63
				311>101	15	100
				311>97	15	13
				255>87	15	5
				255>129	10	3
Methyl cis-9-hexadecenoate	C16:1cMe	1120-25-8	24.710 (24.50-24.95)	268*>155	10	8
				268*>185	10	7
				194>96	10	77
				194>82	10	100
				141>109	10	20
				141>57	10	17

Methyl trans-9-octadecenoate	C18:1tMe	112-62-9	26.878 (26.80-27.45)	296*>213	10	8
				296*>141	10	37
				264>98	10	100
				264>111	10	69
				222>96	10	77
				222>82	15	99
Methyl cis-9-octadecenoate	C18:1cMe	2777-58-4	27.018 (26.80-27.45)	296*>213	10	8
				296*>141	10	34
				264>98	10	100
				264>111	10	70
				222>96	10	76
				222>82	15	98
Methyl cis,cis-9,12-octadecadienoate	C18:2cMe	112-63-0	27.684 (27.45-28.00)	294*>96	15	17
				294*>178	5	5
				123>81	10	100
				123>67	15	62
				150>107	10	44
				150>121	10	28
Methyl cis,cis,cis-6,9,12-octadecatrienoate	C18:3c6Me	16326-32-2	28.167 (28.00-28.50)	292*>94	10	5
				292*>108	20	3
				121>106	10	51
				121>92	10	86
				108>79	10	100
				108>67	15	23
Methyl cis,cis,cis Methyl cis,cis,cis-9,12,15-octadecatrienoate	C18:3c9Me	301-00-8	28.556 (28.50-28.80)	292*>94	20	3
				292*>108	15	1
				136>81	15	7
				136>121	10	7
				108>79	5	100
				108>67	10	18
Methyl cis,cis,cis,cis,cis-5,8,11,14,17-eicosapentaenoate	C20:5cMe	2734-47-6	31.389 (31.35-31.78)	316*>135	15	1
				316*>94	30	1
				133>91	15	43
				133>105	10	47
				108>79	5	100
				108>67	15	12

3 Ion ratio stability

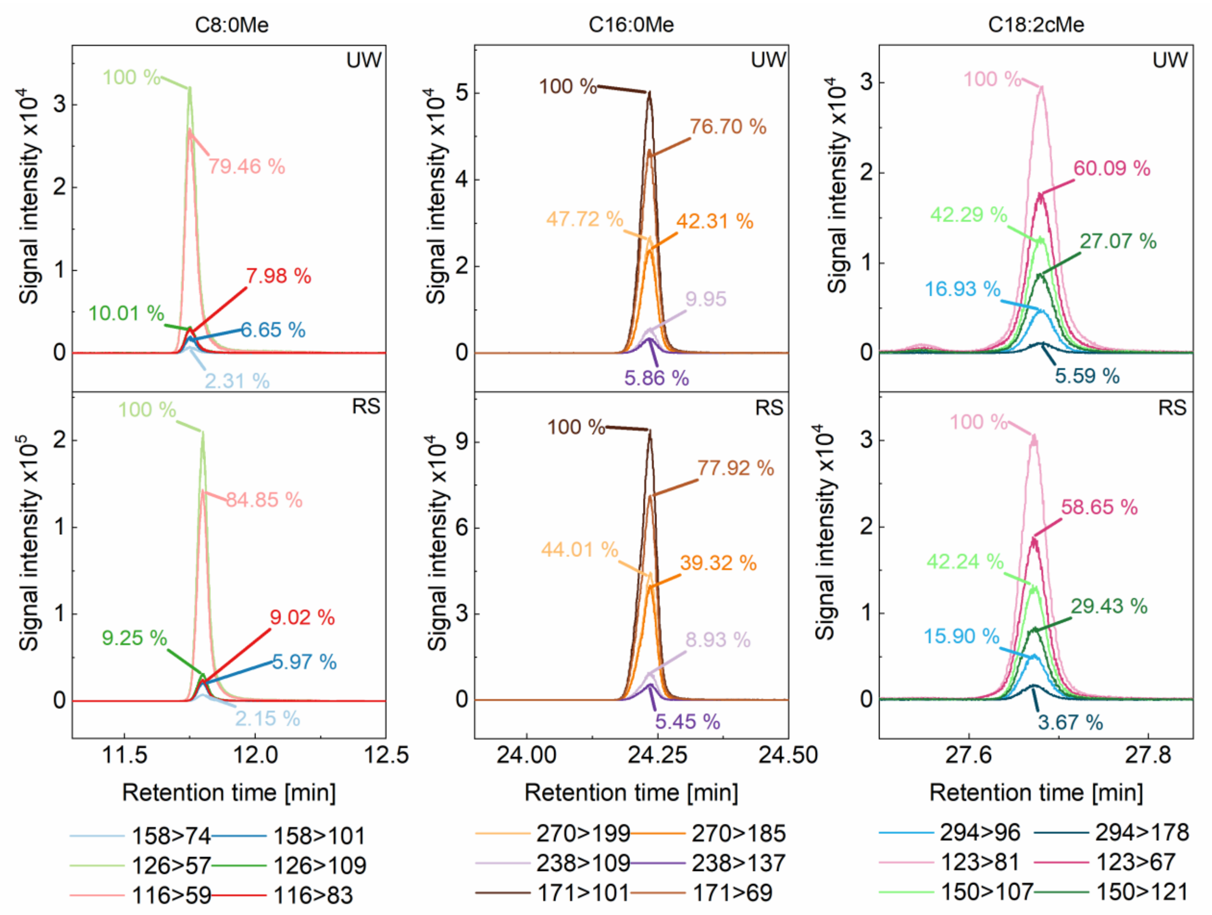

Fig. S1 Ion ratio stability in ultrapure water (UW) spiked in 2-3 µg L^{-1} and spiked real sample (RS) in 0.5-1.5 µg L^{-1} examplarily shown for C8:0Me, C16:0Me and C18:2cMe. Spiking solution: varied concentration FAME Mix

4 Theoretical extracted fractions

The partitioning of analytes in the three phases (headspace, sample, and polymer sorption phase of SPME arrow) under equilibrium conditions is described through the mass balance with the initial mass of the sample m_0, the mass in the aqueous phase m_w, the mass in the gaseous phase m_a and the mass extracted by the fiber m_f (Equation 1).

$$m_0 = m_w + m_a + m_f \quad (1)$$

First, the two equilibria describing the distribution of analytes between the water phase, gas phase, and fiber material are established (Equations 2 and 3). From this, the distribution coefficients between the water phase to gas phase K_{aw} and gas phase to fiber K_{fa} can be established.

$$Analyte\ (aq) \rightleftharpoons Analyte\ (g) \qquad K_{aw} = \frac{[Analyte\ (a)]}{[Analyte\ (w)]} \qquad 2$$

$$Analyte\ (g) \rightleftharpoons Analyte\ (f) \qquad K_{fa} = \frac{[Analyte\ (f)]}{[Analyte\ (a)]} \qquad 3$$

If the equilibria are combined and balanced, the equilibrium for the distribution between water phase to fiber is obtained and the corresponding distribution coefficient K_{fw} can be calculated as follows:

$$Analyte\ (aq) \rightleftharpoons Analyte\ (f) \qquad K_{fw} = \frac{[Analyte\ (f)]}{[Analyte\ (w)]} = K_{aw} \cdot K_{fa} \qquad 4$$

The volume ratio r_{fw} of SPME arrow fiber volume (3.8 µL) to water phase (10 mL) gives:

$$r_{fw} = \frac{V_f}{V_w} = \frac{3.8 \cdot 10^{-6} L}{0.01\ L} = 3.8 \cdot 10^{-4} \qquad 5$$

Theoretical extracted analyte fractions with an SPME arrow fiber (PDMS polymer sorption phase) f_f were determined by Equation 6.

$$f_f = \frac{1}{1 + K_{wf} \cdot r_{wf} + K_{af} \cdot r_{af}} \qquad 6$$

The partitioning constants between air and water K_{aw} and PDMS and air K_{fa} were calculated using the online database for polyparameter linear free energy relationships (pp-LFERs) from the UFZ Helmholz center for environmental research [2] by using equation 7 [3] and equation 8 [4] with experimentally determined pp-LFER descriptors for polarizability/dipolarity S, solute hydrogen bond acidity A, solute hydrogen bond basicity B; McGowan's molar volume V, logarithmic gas-hexane partition coefficient L, and the excess molar refraction E. [5]

$$\log K_{wa} = -0.59 + 2.07\ S + 3.67\ A + 4.87\ B - 2.55\ V + 0.48\ L \qquad 7$$

$$\log K_{fa} = -0.04 + 0.54\ S + 1.14\ A + 0.58\ B + 0.79\ L + 0.01\ E \qquad 8$$

For the reversed equilibrium, the following applies:

$$\log K_{1,2} = -\log\ (K_{2,1}) \qquad 9$$

The temperature-dependent distribution constants for saturated FAMEs were determined using van't Hoffs equation

$$\log \frac{K_1}{K_2} = -\frac{\Delta H_r^0}{2.3\ R} \cdot \left(\frac{1}{T_1} - \frac{1}{T_2}\right) \qquad 10$$

Standard enthalpies of reaction ΔH_r^0 were calculated with standard formation enthalpies for the liquid $\Delta H_f^0(l)$ and gaseous $\Delta H_f^0(g)$ states were taken from CRC Lide [6].

Table S3 Calculated theoretical extracted equilibrium fractions f_f and distribution constants $log\,K_{wa}$, $log\,K_{fw}$ and $log\,K_{fa}$ from literature data. LSER descriptors were taken from the database from the UFZ Helmholz center for environmental research [2]. S: polarizability/dipolarity; A: solute hydrogen bond acidity; B: solute hydrogen bond basicity; V: McGowan's molar volume; L: logarithmic gas-hexane partition coefficient; E: excess molar refraction

FAME	S	A	B	V	L	E	$\log K_{wa}$ (25 °C)	$\log K_{fw}$ (25 °C, PDMS)	$\log K_{fa}$ (25 °C, PDMS)	f_f [%]
C6:0Me	0.6	0	0.45	1.1693	3.874	0.08	1.73	1.88	3.61	2.8
C7:0Me	0.6	0	0.45	1.3102	4.356	0.08	1.59	2.39	3.99	8.5
C8:0Me	0.6	0	0.45	1.4511	4.838	0.07	1.47	2.91	4.37	22.6
C9:0Me	0.6	0	0.45	1.592	5.321	0.06	1.34	3.41	4.75	48.3
C10:0Me	0.6	0	0.45	1.7329	5.803	0.05	1.21	3.93	5.13	74.9
C11:0Me	0.63	0	0.45	1.8738	6.296	0.05	1.15	4.38	5.54	89.7
C12:0Me	0.6	0	0.45	2.0147	6.767	0.04	0.95	4.93	5.89	96.7
C13:0Me	0.64	0	0.45	2.1556	7.271	0.04	0.92	5.39	6.31	98.8
C14:0Me	0.6	0	0.45	2.2965	7.731	0.03	0.7	5.95	6.65	99.6
C15:0Me	0.68	0	0.45	2.4374	8.242	0.04	0.75	6.36	7.1	99.9
C16:0Me	0.6	0	0.45	2.5783	8.695	0.02	0.44	6.97	7.41	100.0
C17:0Me	0.6	0	0.45	2.7192	9.177	0.01	0.31	7.48	7.79	100.0
C18:0Me	0.6	0	0.45	2.8601	9.659	0.01	0.19	7.99	8.18	100.0
C19:0Me	0.6	0	0.45	3.001	10.238	0.01	0.11	8.50	8.63	100.0
C20:0Me	0.6	0	0.45	3.1419	10.75	0.00	-0.01	9.01	9.04	100.0
C18:1cMe	0.6	0	0.62	2.8171	9.76	0.15	1.17	7.22	8.36	100.0
C18:2cMe	0.64	0	0.73	2.7741	9.83	0.30	1.93	6.65	8.5	99.9
C18:3c9Me	0.75	0	0.83	2.7311	9.93	0.41	2.81	5.99	8.7	99.7

5 Single extraction profiles obtained by extraction time optimization

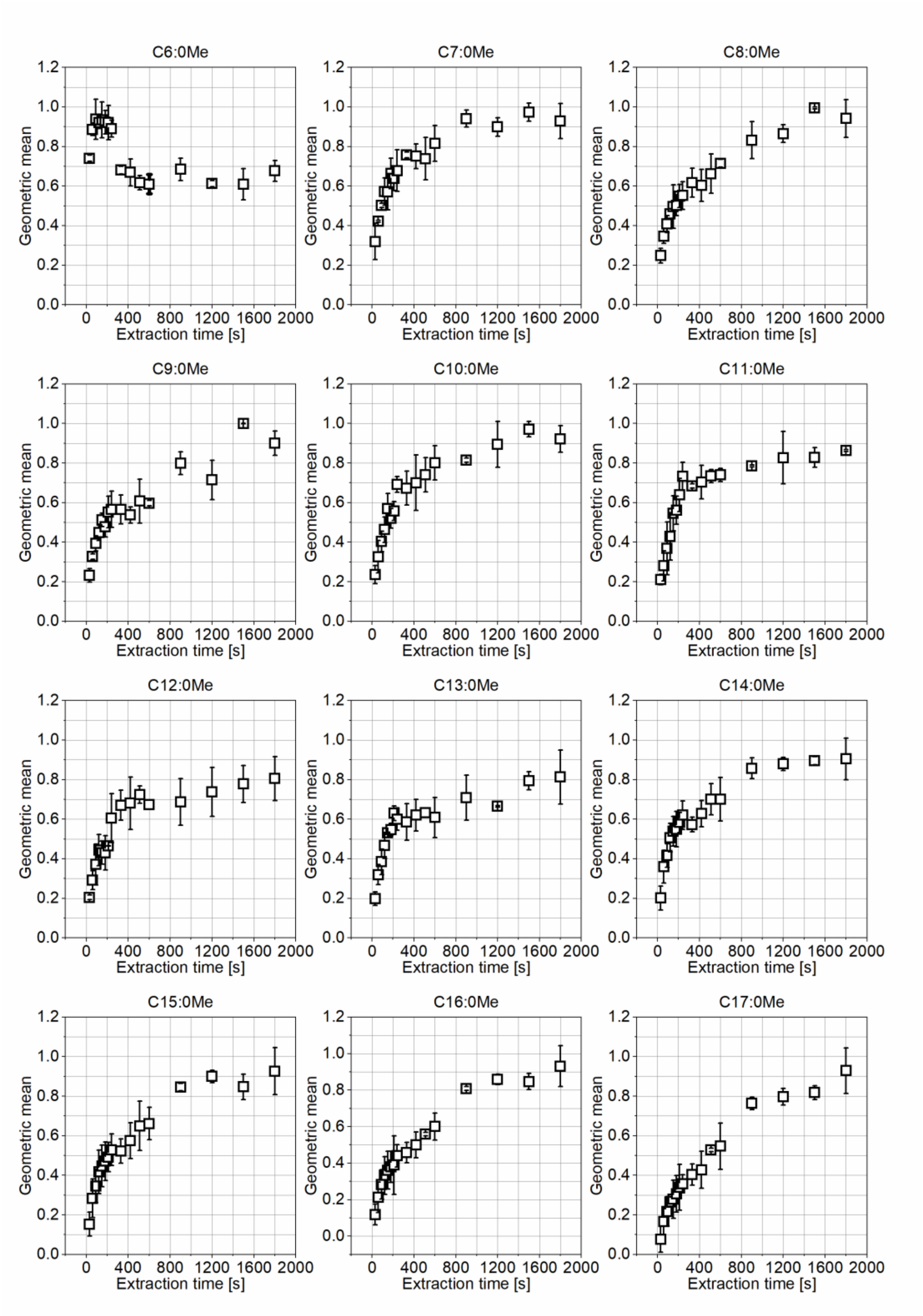

Fig. S2 Optimization of extraction time for C6:0Me, C7:0Me, C8:0Me, C9:0Me, C10:0Me, C11:0Me, C12:0Me, C13:0Me, C14:0Me, C15:0Me, C16:0Me and C17:0Me. Experimental conditions: n = 3; Sample: FAME mix with varying concentrations 1:400,000 diluted in bidistilled water; Extraction parameters: stirring rate: 1500 rpm; DVB-PDMS, 70 °C, pH 2, varying extraction time: 30, 60, 90, 120, 150, 180, 210, 240, 330, 420, 510, 600, 900, 1200, 1500, 1800 s

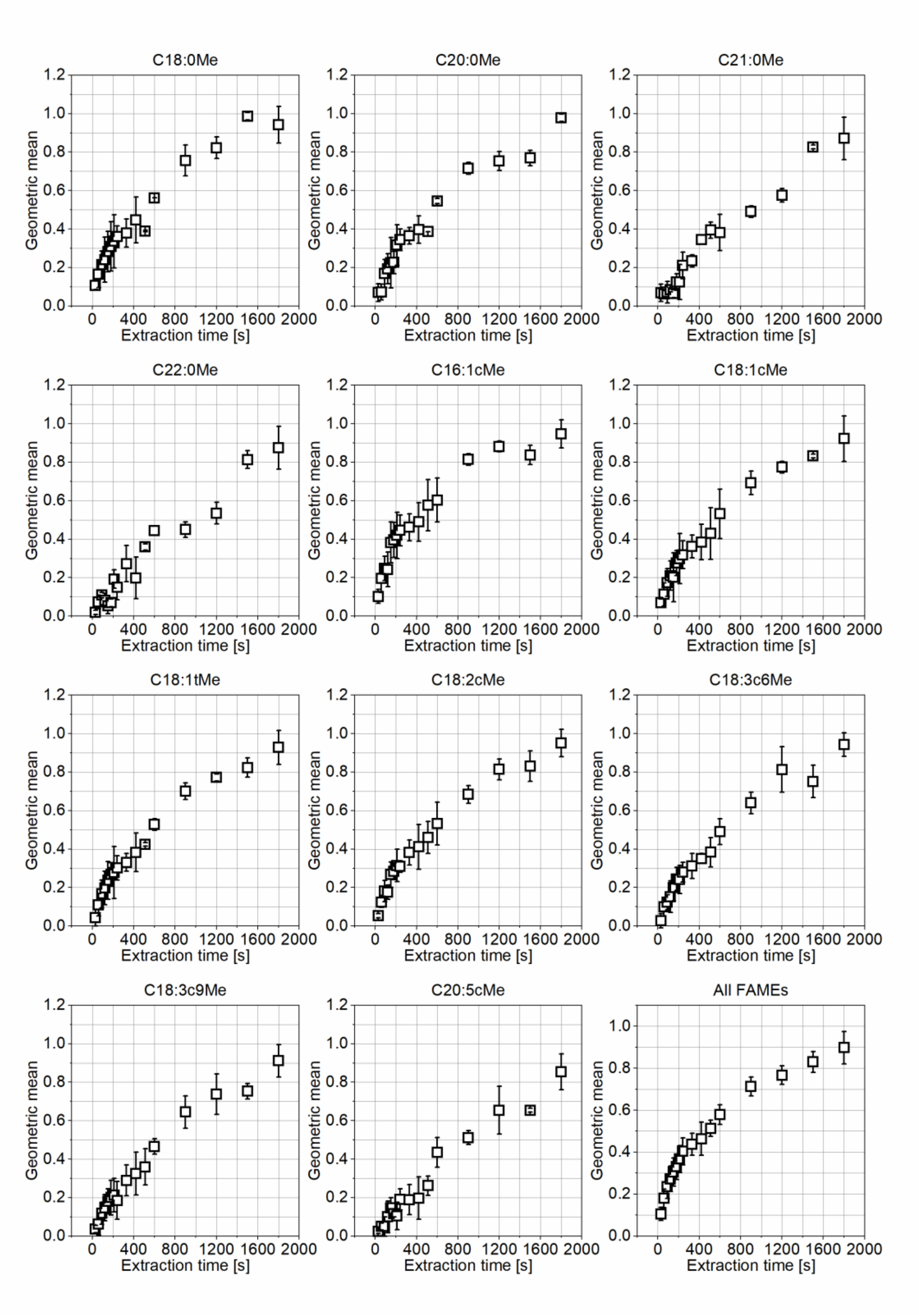

Fig. S3 Optimization of extraction time for C18:0Me, C20:0Me, C21:0Me, C22:0Me, C16:1cMe, C18:1tMe, C18:1cMe, C18:2cMe, C18:3c6Me, C18:3c9Me, C20:5cMe and for all FAMEs. Experimental conditions: n = 3; Sample: FAME mix with varying concentrations 1:400,000 diluted in bidistilled water; Extraction parameters: stirring rate: 1500 rpm; DVB-PDMS, 70 °C, pH 2, varying extraction time: 30, 60, 90, 120, 150, 180, 210, 240, 330, 420, 510, 600, 900, 1200, 1500, 1800 s

6 Calculation of hydrolysis half-life

To explain the shape of the curve for the fit of the extraction pH optimization, pH-dependent FAME hydrolysis has to be taken into account. Therefore the hydrolysis rate constant k_h and the hydrolysis half-live $t_{0.5}$, where 50 % of FAMEs are hydrolyzed, were calculated. Pseudo-first order rate constants at 25 °C for the acid, neutral, and base-catalyzed hydrolysis of saturated FAMEs were taken from Rayne *et al.* [7].

$$k_h = k_A \cdot [H^+] + k_N + k_B \cdot [OH^-] \qquad 11$$

$$t_{0.5} = \frac{\ln\left(\frac{1}{0.5}\right)}{k_h} \qquad 12$$

As the pseudo-first-order rate constants were the same for C6:0Me-C18:0Me, they are independent of chain length and here only shown exemplarily for C6:0Me.

7 Observation of droplet formation on SPME arrow surface

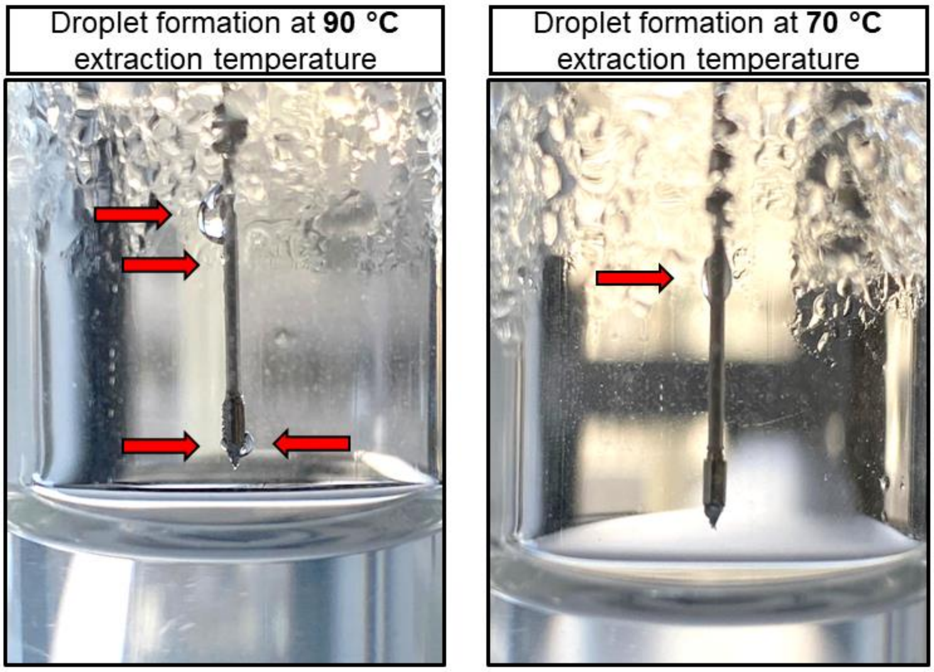

Fig. S4 Observed increased droplet formation at SPME arrow surface at 90 °C compared to 70 °C extraction temperature. Droplets are indicated by red arrows

8 Calibration plots and linear regression functions

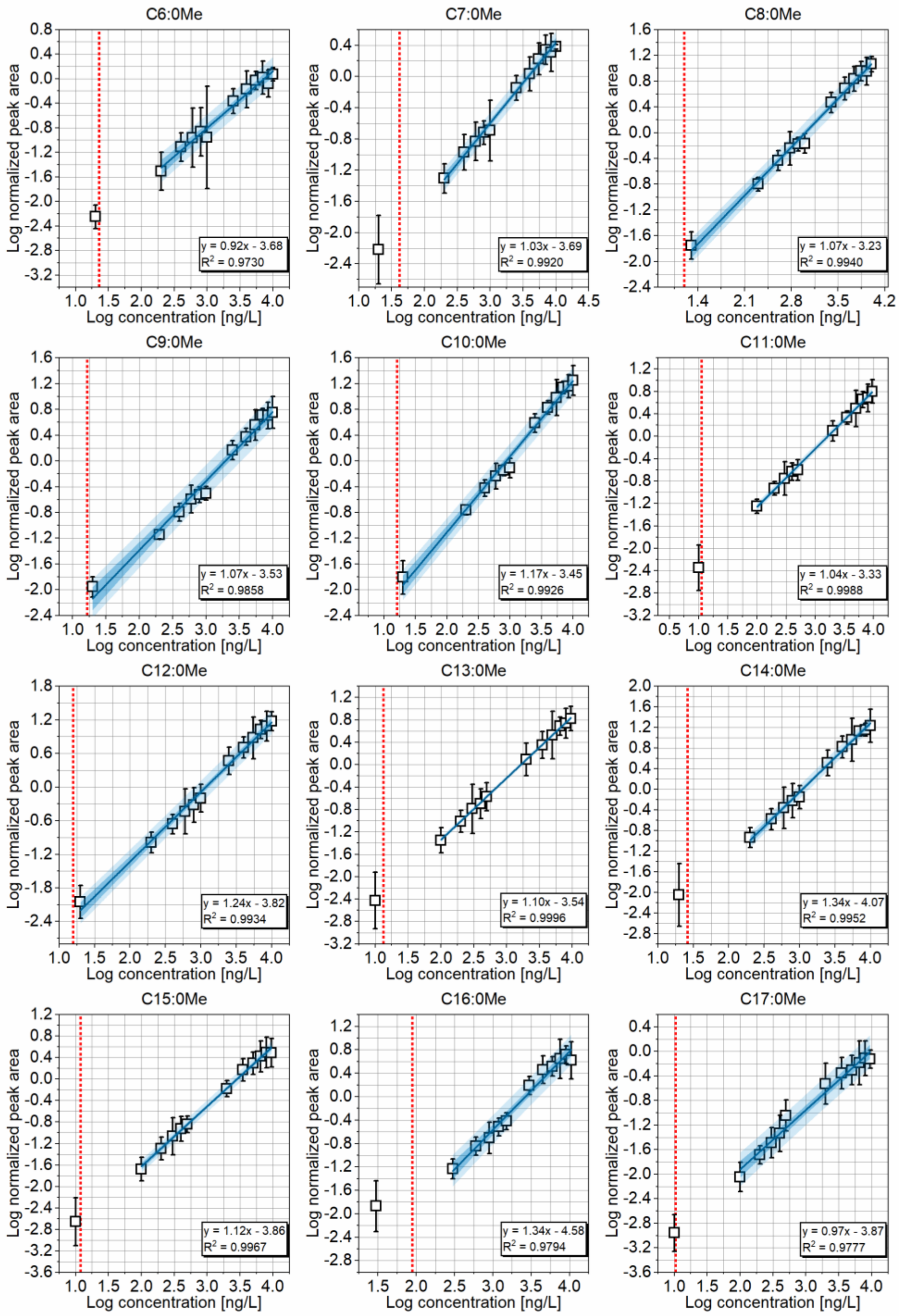

Fig. S5 Double logarithmic visualized calibration plots for C6:0Me, C7:0Me, C8:0Me, C9:0Me, C10:0Me, C11:0Me, C12:0Me, C13:0Me, C14:0Me, C15:0Me, C16:0Me and C17:0Me. Light blue: 95 % prognosis range, dark blue: 95 % confidence range, red dotted line: MDL. Peak area was normalized to the internal standard C17:0dMe. Experimental parameters: n = 7; Sample: FAME mix with varying concentrations 1:400,000 diluted in bidistilled water; Extraction parameters: pH 2, DVB-PDMS, 70 °C, stirring rate: 1500 rpm, extraction time: 1200 s

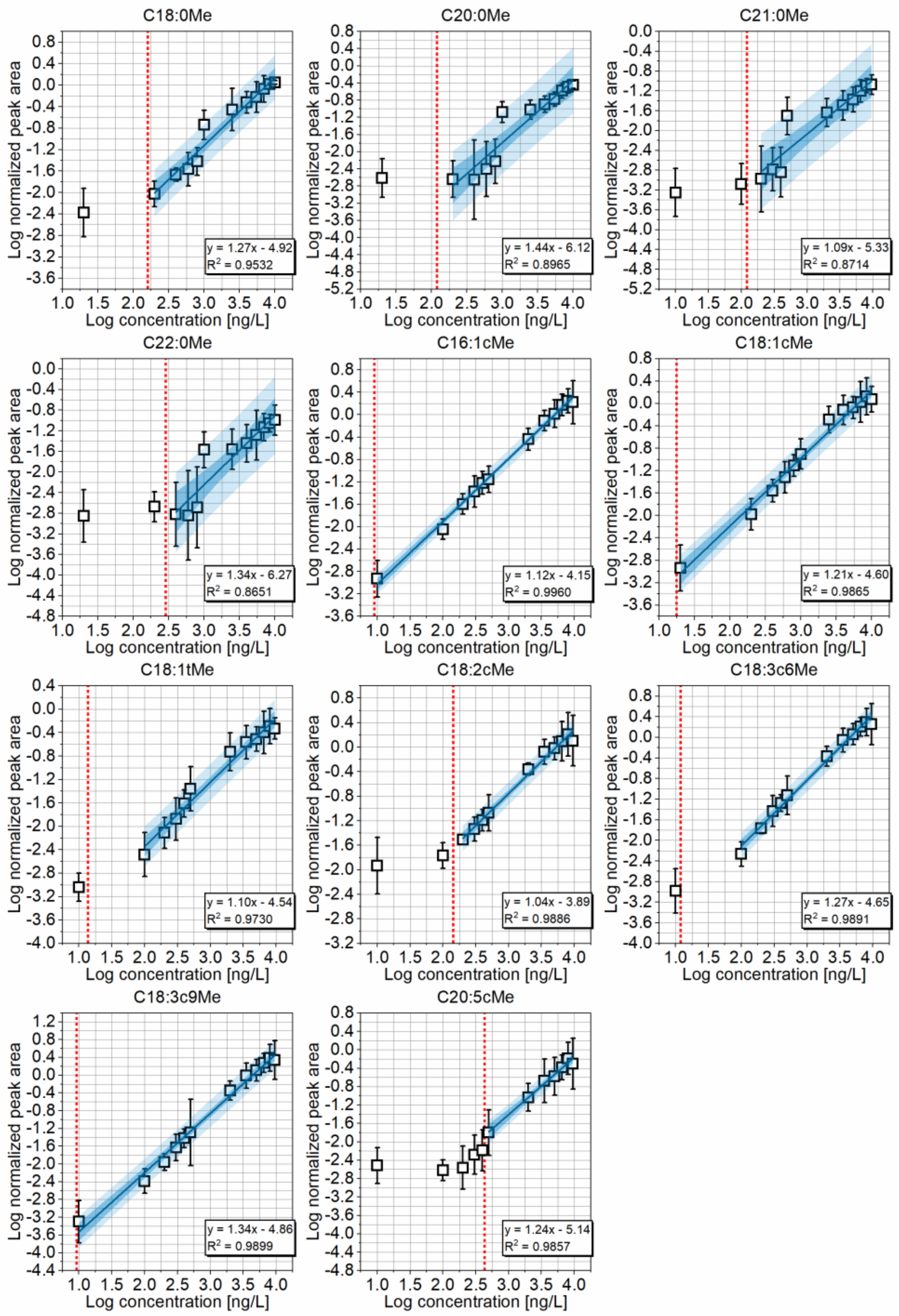

Fig. S6 Double logarithmic visualized calibration plots for C18:0Me, C20:0Me, C21:0Me, C22:0Me, C16:1cMe, C18:1tMe, C18:1cMe, C18:2cMe, C18:3c6Me, C18:3c9Me, C20:5cMe. Light blue: 95 % prognosis range, dark blue: 95 % confidence range, red dotted line: MDL. Peak area was normalized to the internal standard C17:0dMe. Experimental parameters: n = 7; Sample: FAME mix with varying concentrations 1:400,000 diluted in bidistilled water; Extraction parameters: pH 2, DVB-PDMS, 70 °C, stirring rate: 1500 rpm, extraction time: 1200 s

Table S4 Linear regression functions with slope m and y-intercept b from both calibrations, 1 and 2, and linearized extraction efficiency plots with correlation coefficient (m_E, b_E, R^2_E)

FAME	m_1	b_1	m_2	b_2	m_E	b_E	R^2_E
C6:0Me	$1.74\cdot10^{-4}$	$1.73\cdot10^{-3}$	$1.04\cdot10^{-4}$	$1.70\cdot10^{-1}$	$-1.4\cdot10^{-1}$	$2.0\cdot10^{-1}$	0.9231
C7:0Me	$2.39\cdot10^{-4}$	$3.80\cdot10^{-3}$	$2.46\cdot10^{-4}$	$1.18\cdot10^{-1}$	$-4.3\cdot10^{-1}$	$7.1\cdot10^{-1}$	0.9912
C8:0Me	$8.72\cdot10^{-4}$	$4.59\cdot10^{-3}$	$1.17\cdot10^{-3}$	$-6.92\cdot10^{-3}$	$-8.9\cdot10^{-1}$	$8.3\cdot10^{-1}$	0.9841
C9:0Me	$3.86\cdot10^{-4}$	$3.08\cdot10^{-3}$	$6.19\cdot10^{-4}$	$-2.65\cdot10^{-2}$	$-6.6\cdot10^{-1}$	$-9.5\cdot10^{-1}$	0.8100
C10:0Me	$9.23\cdot10^{-4}$	$-4.71\cdot10^{-4}$	$1.88\cdot10^{-3}$	$-7.90\cdot10^{-1}$	$-7.0\cdot10^{-1}$	$-1.52\cdot10^{0}$	0.7857
C11:0Me	$5.94\cdot10^{-4}$	$-2.09\cdot10^{-3}$	$6.60\cdot10^{-4}$	$-9.59\cdot10^{-2}$	$-6.4\cdot10^{-1}$	$-1.72\cdot10^{0}$	0.7581
C12:0Me	$6.15\cdot10^{-4}$	$-1.33\cdot10^{-2}$	$1.60\cdot10^{-3}$	$-1.05\cdot10^{0}$	$-6.3\cdot10^{-1}$	$-1.57\cdot10^{0}$	0.7920
C13:0Me	$5.24\cdot10^{-4}$	$-3.85\cdot10^{-3}$	$7.19\cdot10^{-4}$	$-1.49\cdot10^{-1}$	$-6.5\cdot10^{-1}$	$-1.38\cdot10^{0}$	0.8175
C14:0Me	$7.71\cdot10^{-4}$	$-2.60\cdot10^{-2}$	$1.86\cdot10^{-3}$	$-9.89\cdot10^{-1}$	$-6.4\cdot10^{-1}$	$-8.2\cdot10^{-1}$	0.8914
C15:0Me	$3.03\cdot10^{-4}$	$-5.63\cdot10^{-3}$	$3.56\cdot10^{-4}$	$9.75\cdot10^{-2}$	$-5.7\cdot10^{-1}$	$-3.2\cdot10^{-1}$	0.9274
C16:0Me	$2.45\cdot10^{-4}$	$-5.26\cdot10^{-3}$	$4.79\cdot10^{-4}$	$2.49\cdot10^{-1}$	$-4.3\cdot10^{-1}$	$-2.9\cdot10^{-1}$	0.9941
C17:0dMe	-	-	-	-	$-6.9\cdot10^{-1}$	$2.6\cdot10^{-1}$	0.9393
C17:0Me	$1.17\cdot10^{-4}$	$-1.63\cdot10^{-3}$	$7.48\cdot10^{-5}$	$1.27\cdot10^{-1}$	$-4.5\cdot10^{-1}$	$-2.7\cdot10^{-1}$	0.9464
C18:0Me	$4.36\cdot10^{-5}$	$2.48\cdot10^{-3}$	$1.09\cdot10^{-4}$	$6.08\cdot10^{-2}$	$-2.3\cdot10^{-1}$	$-5.0\cdot10^{-1}$	0.9964
C20:0Me	$4.51\cdot10^{-6}$	$1.57\cdot10^{-3}$	$3.40\cdot10^{-5}$	$3.30\cdot10^{-2}$	$-1.2\cdot10^{-1}$	$-3.4\cdot10^{-1}$	0.8054
C21:0Me	$2.63\cdot10^{-6}$	$5.79\cdot10^{-4}$	$8.10\cdot10^{-6}$	$8.97\cdot10^{-3}$	$-2.5\cdot10^{-1}$	$-1.8\cdot10^{-1}$	0.8570
C22:0Me	$3.18\cdot10^{-7}$	$1.58\cdot10^{-3}$	$9.11\cdot10^{-6}$	$7.94\cdot10^{-3}$	$-3.0\cdot10^{-1}$	$-5.0\cdot10^{-1}$	0.8199
C16:1cMe	$1.56\cdot10^{-4}$	$-3.78\cdot10^{-3}$	$1.98\cdot10^{-4}$	$3.66\cdot10^{-2}$	$-6.9\cdot10^{-1}$	$-3.3\cdot10^{-1}$	0.9393
C18:1tMe	$5.86\cdot10^{-5}$	$-1.88\cdot10^{-3}$	$4.89\cdot10^{-5}$	$6.92\cdot10^{-2}$	$-4.5\cdot10^{-1}$	-0.29	0.9464
C18:1cMe	$9.84\cdot10^{-5}$	$-6.62\cdot10^{-3}$	$1.22\cdot10^{-4}$	$1.63\cdot10^{-1}$	$-2.3\cdot10^{-1}$	0.26	0.9964
C18:2cMe	$1.38\cdot10^{-4}$	$6.20\cdot10^{-3}$	$1.51\cdot10^{-4}$	$1.68\cdot10^{-1}$	$-1.2\cdot10^{-1}$	-0.27	0.8409
C18:3c6Me	$1.39\cdot10^{-4}$	$-5.38\cdot10^{-3}$	$2.15\cdot10^{-4}$	$5.88\cdot10^{-2}$	$-2.5\cdot10^{-1}$	0.36	0.9939
C18:3c9Me	$9.68\cdot10^{-5}$	$-4.11\cdot10^{-3}$	$2.69\cdot10^{-4}$	$-1.68\cdot10^{-2}$	$-3.0\cdot10^{-1}$	0.45	0.9939
C20:5cMe	$1.01\cdot10^{-5}$	$1.96\cdot10^{-3}$	$6.57\cdot10^{-5}$	$-2.21\cdot10^{-2}$	$-5.4\cdot10^{-1}$	0.33	0.9887

9 Depletion curves obtained by extraction efficiency experiments

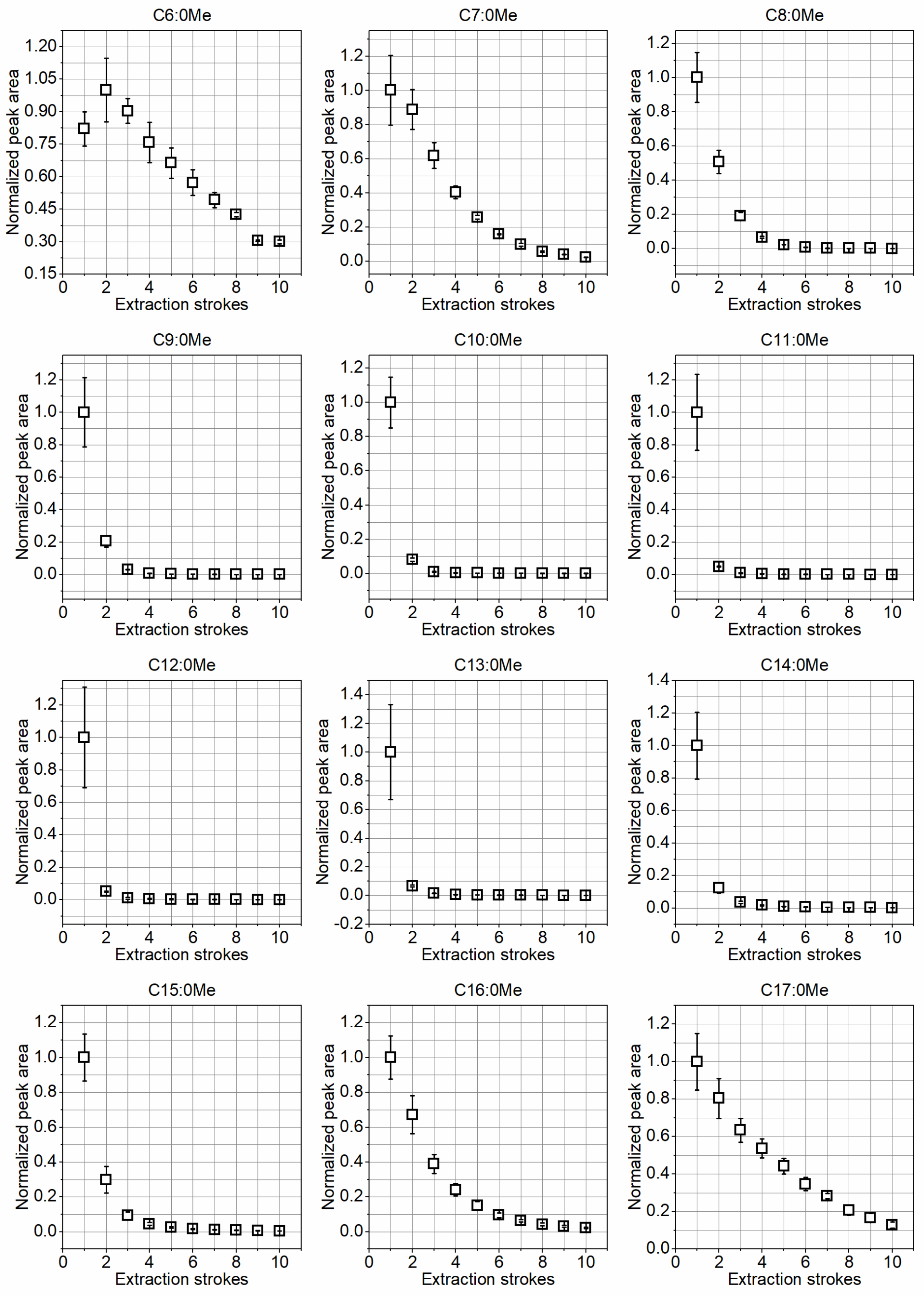

Fig. S7 Depletion curve method plots for determination of extraction efficiency for C6:0Me, C7:0Me, C8:0Me, C9:0Me, C10:0Me, C11:0Me, C12:0Me, C13:0Me, C14:0Me, C15:0Me, C16:0Me and C17:0Me. Peak area was normalized to the highest value. Experimental parameters: n = 7; Sample: FAME mix with varying concentrations 1:400,000 diluted in bidistilled water; Extraction parameters: pH 2, DVB-PDMS, 70 °C, stirring rate: 1500 rpm, extraction time: 1200 s, 10 extraction strokes

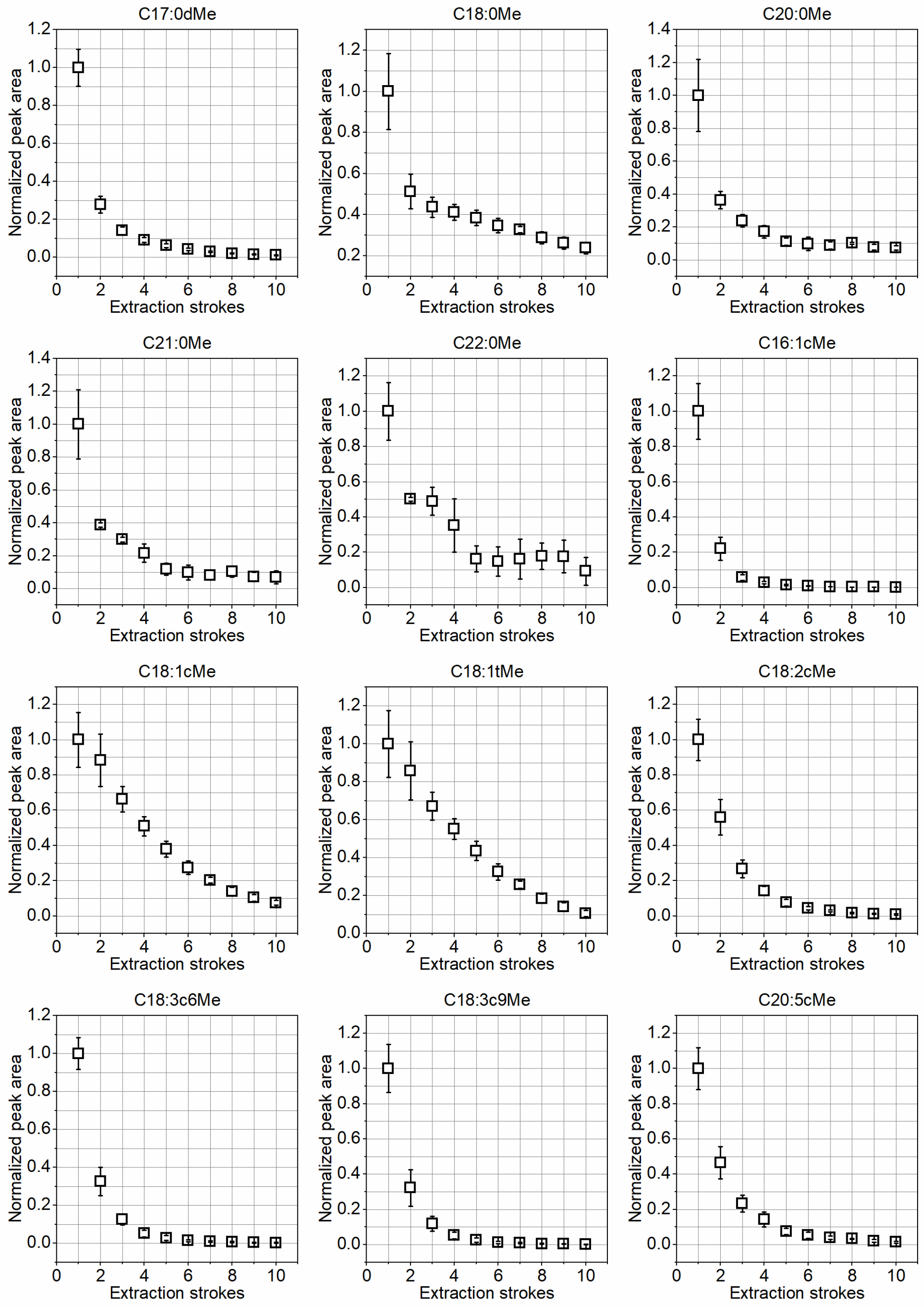

Fig. S8 Depletion curve method plots for determination of extraction efficiency for C18:0Me, C20:0Me, C21:0Me, C22:0Me, C16:1cMe, C18:1tMe, C18:1cMe, C18:2cMe, C18:3c6Me, C18:3c9Me, C20:5cMe. Peak area was normalized to the highest value. Experimental parameters: n = 7; Sample: FAME mix with varying concentrations 1:400,000 diluted in bidistilled water; Extraction parameters: pH 2, DVB-PDMS, 70 °C, stirring rate: 1500 rpm, extraction time: 1200 s, 10 extraction strokes

References

1. Hartig C. Rapid identification of fatty acid methyl esters using a multidimensional gas chromatography-mass spectrometry database. J Chromatogr A. 2008;1177(1):159-69; https://doi.org/10.1016/j.chroma.2007.10.089.
2. Ulrich N, Endo S, Brown TN, Watanabe N, Bronner G, Abraham MH, et al. UFZ-LSER database v 3.2.1. 2017
3. Goss KU. Predicting the equilibrium partitioning of organic compounds using just one linear solvation energy relationship (LSER). Fluid Phase Equilibr. 2005;233(1):19-22; https://doi.org/10.1016/j.fluid.2005.04.006.
4. Sprunger L, Proctor A, Acree WE, Abraham MH. Characterization of the sorption of gaseous and organic solutes onto polydimethyl siloxane solid-phase microextraction surfaces using the Abraham model. J Chromatogr A. 2007;1175(2):162-73; https://doi.org/10.1016/j.chroma.2007.10.058.
5. Abraham MH, Andonian-Haftvan J, Whiting GS, Leo A, Taft RS. Hydrogen bonding. Part 34. The factors that influence the solubility of gases and vapours in water at 298 K, and a new method for its determination. J Chem Soc. 1994(8):1777-91; https://doi.org/10.1039/P29940001777.
6. Haynes WM. CRC Handbook of Chemistry and Physics; 2017.
7. Rayne S, Forest K. Carboxylic acid ester hydrolysis rate constants for food and beverage aroma compounds. Flavour Frag J. 2016;31(5):385-94; https://doi.org/10.1002/ffj.3327.
